# Membrane Drug Transporters and Chemoresistance in Human Pancreatic Carcinoma

**DOI:** 10.3390/cancers3010106

**Published:** 2010-12-30

**Authors:** Wolfgang Hagmann, Ralf Faissner, Martina Schnolzer, Matthias Lohr, Ralf Jesnowski

**Affiliations:** 1 Clinical Cooperation Unit of Molecular Gastroenterology, DKFZ, Im Neuenheimer Feld 280, D-69120 Heidelberg, Germany; E-Mails: ralf.faissner@web.de (R.F.); matthias.lohr@ki.se (M.L.); Ralf.Jesenofsky@medma.uni-heidelberg.de (R.J.); 2 Functional Proteome Analysis, DKFZ, Im Neuenheimer Feld 280, D-69120 Heidelberg, Germany; E-Mail: m.schnoelzer@dkfz.de; 3 Department of Surgical Gastroenterology, CLINTEC, K53, Karolinska Institute, SE-14186 Stockholm, Sweden; 4 Department of Medicine II, Medical Faculty of Mannheim, University of Heidelberg, Theodor-Kutzer-Ufer 1-3, D-68167 Mannheim, Germany

**Keywords:** ABC transporters, chemoresistance, pancreatic cancer, gemcitabine, 5-fluorouracil, multidrug resistance proteins, S100A4, OATPs, nucleoside transporters, RNA interference

## Abstract

Pancreatic cancer ranks among the tumors most resistant to chemotherapy. Such chemoresistance of tumors can be mediated by various cellular mechanisms including dysregulated apoptosis or ineffective drug concentration at the intracellular target sites. In this review, we highlight recent advances in experimental chemotherapy underlining the role of cellular transporters in drug resistance. Such contribution to the chemoresistant phenotype of tumor cells or tissues can be conferred both by uptake and export transporters, as demonstrated by *in vivo* and *in vitro* data. Our studies used human pancreatic carcinoma cells, cells stably transfected with human transporter cDNAs, or cells in which a specific transporter was knocked down by RNA interference. We have previously shown that 5-fluorouracil treatment affects the expression profile of relevant cellular transporters including multidrug resistance proteins (MRPs), and that MRP5 (ABCC5) influences chemoresistance of these tumor cells. Similarly, cell treatment with the nucleoside drug gemcitabine or a combination of chemotherapeutic drugs can variably influence the expression pattern and relative amount of uptake and export transporters in pancreatic carcinoma cells or select for pre-existing subpopulations. In addition, cytotoxicity studies with MRP5-overexpressing or MRP5-silenced cells demonstrate a contribution of MRP5 also to gemcitabine resistance. These data may lead to improved strategies of future chemotherapy regimens using gemcitabine and/or 5-fluorouracil.

## 1. Introduction

Pancreatic cancer is the fourth most common cause of cancer-related death in the Western world, with an estimated 36,800 deaths in 2010 in the United States [1]. Because early diagnostic markers are missing and due to a lack of early symptoms, less than 20% of the diagnosed pancreatic cancer patients are considered for a potentially curative resection, and many patients who undergo tumor resection suffer from recurrences and die within the first three years [2]. Despite chemotherapy, the median survival time of patients with advanced disease is only about six months and the five-year-survival rate is below 4% [3], mostly because of an almost complete resistance against established radio/chemotherapies.

Such resistance of human cells to the cytotoxic action of chemotherapeutic drugs can be the result of various mechanisms, of which three major mechanisms have been identified: a reduced uptake of the drugs into the target cells; alterations within the cells, like altered metabolism of the drugs, increased capacity to repair DNA or reduced apoptosis; and an increased efflux of the drugs from the cells [4]. Another mechanism related to chemresistance is the so-called cell-adhesion mediated drug resistance (CAM-DR) [5]. Earlier, pancreatic cancer was usually treated with 5-fluorouracil (5-FU). Today, gemcitabine chemotherapy is the standard of care for advanced and metastatic pancreatic cancer, however, 5-FU and its orally available analogue capecitabine are both acknowledged for first line therapy after surgery [6] and second-line therapy in advanced pancreatic cancer [7]. All drugs are nucleoside analogues; therefore, some of the above mentioned resistance mechanisms will be exemplified with the drugs 5-FU or gemcitabine. Water soluble drugs such as nucleoside analogues or cisplatin require specialized membrane transporters to efficiently enter the cell. The cytidine analog gemcitabine (2′-,2′-difluorodeoxycytidine) is taken up into cells primarily through the specialized concentrative or equilibrative nucleoside transporters CNT1 (gene symbol: *SLC28A1*), CNT3 (*SLC28A3*), ENT1 (*SLC29A1*) and ENT2 (*SLC29A2*) [8-11]. In pancreatic tumor cells, expression of ENT1 or CNT1 has both previously been linked to gemcitabine resistance or sensitivity of pancreatic cancer cells [12-14]. Most importantly, recent studies showed that expression levels of *ENT1* and *CNT3* are predictive for patient survival times after gemcitabine treatment [15,16].

Within the cells, gemcitabine has to be activated by phosphorylation to the active triphosphate metabolite dFdCTP, which then is incorporated into DNA. The enzyme deoxycytidine kinase (DCK) catalyzes the first rate-limiting step in these reactions [17]. Consequently, DCK is associated with prolonged survival of patients after adjuvant gemcitabine therapy for resected pancreatic ductal adenocarcinoma (PDAC) [18], and downregulation of DCK *in vitro* enhances resistance against gemcitabine in pancreatic tumor cells [19]. Moreover, the RNA-binding protein HuR modulates the translation of target mRNAs including *DCK* mRNA, in which case HuR overexpression elevates, and HuR silencing reduces DCK protein expression; thus, HuR expression was also found to be a outcome predictor for patients with resected PDAC during adjuvant gemcitabine therapy [20,21].

Finally, drug efflux from the cell is efficiently mediated by proteins belonging to the ATP-binding cassette (ABC) family of transporters [22-25]. Several members of the multidrug resistance protein family (MRPs), MDR1 P-glycoprotein and the breast cancer resistance protein (BCRP) have been demonstrated to confer chemoresistance or multidrug resistance [22-28]. These ABC transporters belong to the ABCC (MRP–MRP9; gene symbol: *ABCC1*–*ABCC6* and *ABCC10*–*ABCC12*), ABCB (MDR1 P-glycoprotein; symbol *ABCB1*), and ABCG (BCRP; symbol *ABCG2*) families of ABC transporters, respectively. In addition to their natural endogenous substrates, the MRP proteins, MDR1, and BCRP also transport a vast array of cytotoxic substances (for review, see [4,29], and MRP3 (ABCC3), MRP4 (ABCC4), and MRP5 have been demonstrated to confer resistance against chemotherapeutic drugs such as etoposide, 5-fluorouracil (5-FU), and gemcitabine [26,27,30-32]. These transporters, among other ABC transporters, are expressed in normal or diseased human pancreas [33-38]. Conversely, the apparent cellular expression profile of transporters can be altered by chemotherapeutic drugs [26,27,38-40], leading to a cellular phenotype which can also be the result of a drug-induced selection mechanism for pre-existing subpopulations of cells.

Given the importance of ABC transporter expression for chemoresistance, the analysis of regulatory mechanisms resulting in overexpression of these transporters is mandatory to identify new targets, which may help to overcome the resistance phenotype of PDAC. For example, several ABC transporter genes are transcriptionally regulated by nuclear factor (erythroid-derived 2)-like 2 (Nrf2) protein. Overexpression of Nrf2 protein levels in pancreatic cancer cells resulted in increased drug resistance, whereas a reduction in Nrf2 protein decreased drug resistance. These changes in drug resistance or sensitivity were also positively correlated to the expression levels of Nrf2 downstream genes *ABCG2*, *MRP1*, *MRP2* and *MRP4* [41]. Moreover, constitutive activation of the hedgehog (HH) pathway induces chemoresistance in a variety of cancers including PDAC by regulation of ABC transporter expression, like ABCB1 and ABCG2. Interfering with HH activation sensitizes the tumor cells towards the cytotoxic effects of chemotherapy [42-46]. Moreover, a number of studies have demonstrated inhibition of ABC transporters by flavonoids, thus potentially affecting the distribution and cytotoxicity of chemotherapeutic drugs [47,48]. miRNAs, small non-coding RNAs regulating gene expression, have been shown to influence chemoresistance in several cancers. In PDAC, patients with high miR-21 expression had a significantly shorter overall survival, and low miR-21 expression was associated with benefit from adjuvant treatment [49]. *In vitro*, anti-miR-21 increased anticancer drug activity, while pre-miR-21 transfection significantly decreased antiproliferative effects and apoptosis induction by gemcitabine [50,51]. Moreover, hsa-miR-520h was shown to downregulate ABCG2 and to inhibit migration, invasion and side populations (SP) in pancreatic cancer cells [52].

Further, enhanced expression of ABC transporters seems to be characteristic for SP cells with cancer stem cell features [53,54]. These stem cells are more resistant against chemotherapies and may play a key role in tumor progression, recurrence and metastasis [55-58]. A further possible link between transporters and chemoresistance is represented by mucin MUC4. Interestingly, MUC4 is overexpressed in PDAC tissue with no significant expression in normal pancreatic tissue (for review: see [59]). Overexpression of MUC4 in PDAC cell lines was associated with a higher resistance against gemcitabine and treatment of MUC4-overexpressing PDAC cells with gemcitabine resulted in an enrichment of SP cells. In contrast, in MUC4 knockdown PDAC cells, all cells including the SP fraction were responsive to the cytotoxic effects induced by gemcitabine treatment [60]. Our own studies thus aim to link data on transporter expression, chemoresistance, and chemosensitivity, especially regarding gemcitabine treatment, to this complex field of pancreatic cancer cell biology in order to provide new aspects for improved future treatment of this aggressive cancer type.

## 2. Results and Discussion

Endogenous compounds or xenobiotics including chemotherapeutic drugs enter or leave human cells most efficiently via specialized uptake or export transporters localized at the appropriate cellular membrane domain. Thus, the detailed knowledge of the specific transporter expression profile of a certain cell, tissue, or organ is a prerequisite to understand and improve drug delivery and therapeutic effectiveness at the body site in question.

### 2.1. Expression Profile of Uptake Transporters of the OATP Family in Pancreatic Cells

**Figure 1.**
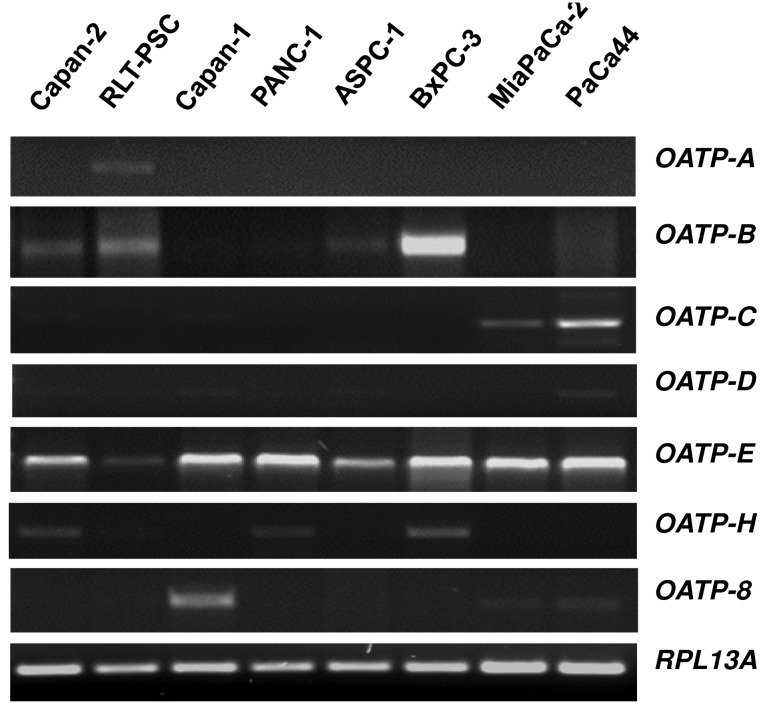
Expression of *OATP* isoform mRNAs in human pancreatic cell lines. RT-PCR was performed with primer pairs specific for the respective transporter isoform or for RPL13A as pancreatic housekeeping gene [66] and analyzed by electrophoresis using 2% agarose gel and ethidium bromide staining. RLT-PSC: Immortalized pancreatic stellate cell line [67]; others are human pancreatic cancer cell lines.

Organic anion transporting polypeptides (OATPs) represent membrane transport proteins mediating the sodium-independent transport of a wide range of amphipathic organic compounds including bile salts, organic dyes, steroid conjugates, thyroid hormones, anionic oligopeptides, drugs and other xenobiotic substances [61]. Whereras some OATPs are apparently selectively expressed in liver [62,63], most OATPs are expressed in multiple tissues [64]. Our novel data on the *OATP* mRNA expression spectrum using a panel of eight different human pancreatic cells showed that among the seven OATP family members studied, only *OATP-E* was expressed in all pancreatic cells (Figure 1), whereas *OATP-F*, *OATP-I*, *and PGT* were not detectable (data not shown). *OATP-B*, *OATP-C* and *OATP-H* were detected in some cell lines, but *OATP-A*, *OATP-D*, and *OATP-8* only in one of these cells. Although our *in vitro* mRNA data do not necessarily reflect the respective cellular OATP protein expression, they may indicate that OATP-E is the main family member of these uptake transporters present in pancreatic cells. OATP-E has been reported to mediate uptake of taurocholate, thyroid hormones, and PGE2 [65]. So far, OATP-E has not been demonstrated to transport chemotherapeutic drugs. Further studies will thus clarify whether OATP-E is also involved in the uptake of chemotherapeutic drugs in pancreatic cells.

### 2.2. mRNA Expression Profile of ABC Transporters of the MRP Family in Pancreatic Cells

**Figure 2.**
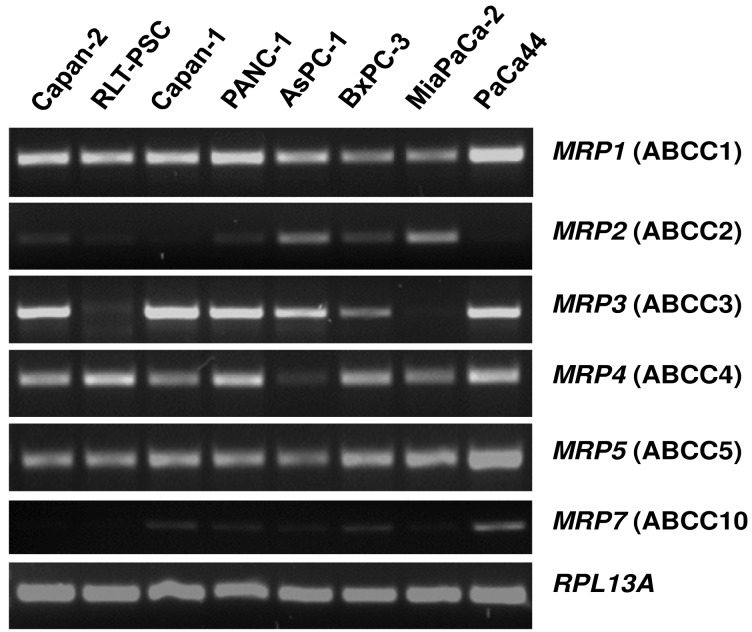
*MRP* mRNA expression in human pancreatic cells. RT-PCR and amplicon analyses were performed as in Figure 1 (adapted from [26] with permission from S. Karger AG).

The mRNA expression profile of MRP family members in human pancreatic carcinoma cell lines (Figure 2) closely resembles the pattern found in normal pancreatic duct cells [26]. *MRP1*, *MRP3*, *MRP4*, and *MRP5* mRNAs are strongly expressed in most of these cancer cells, while *MRP2* and *MRP7* are only weakly expressed (Figure 2), and *MRP6*, *MRP8*, and *MRP9* mRNAs were not detected in any of these pancreatic cancer cell lines (data not shown). For comparison, the human pancreatic stellate cell line, RLT-PSC [67], shows strong expression of *MRP1*, *MRP4*, and *MRP5* mRNA, but very low expression of *MRP2* and *MRP3* mRNA (Figure 2); this finding may be relevant in view of the discussed role of this pancreatic cell type in the development of chronic pancreatitis and pancreatic cancer [68-71].

### 2.3. Altered MRP Expression Profile in 5-FU-Resistant Pancreatic Cancer Cells

**Figure 3.**
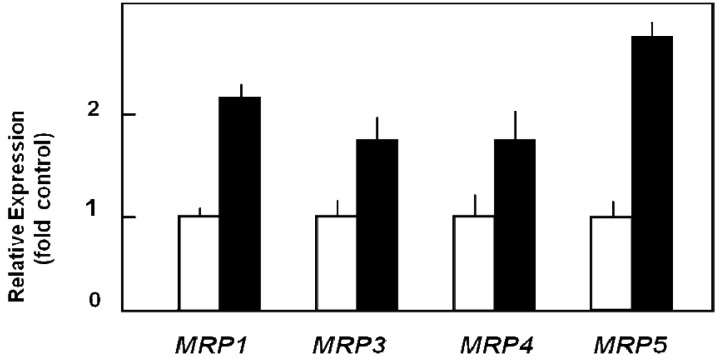
Upregulation of MRP mRNA expression in 5-FU resistant Capan-1 cells. Data indicate relative expression of the respective transporter mRNAs in parental Capan-1 *(white columns)* and in 5-FU resistant Capan-1 cells *(black columns)*, each normalized to *RPL13A* mRNA expression using QRT-PCR analysis and control values set to 1. Mean values and S.D. are from two biological and three technical replicates (adapted from [26]).

Capan-1 cells are characterized by relatively pronounced sensitivity to the cytotoxic action of 5-FU [72,73], but can be adapted to acquire high resistance to this drug by prolonged exposure to it. Such 5-FU-resistant Capan-1 cells, which possess a 27-fold increased LD_50_ value for 5-FU as compared to parental Capan-1 cells [74], show indeed an altered MRP expression profile with about two-fold upregulation of several *MRP* mRNAs (Figure 3) [26]. This increased MRP expression in 5-FU resistant cells is also reflected in the MRP protein level [26]. Most importantly, this elevated ABC transporter protein level in 5-FU resistant cells holds true for the relatively highly expressed MRP3, MRP4, and MRP5 proteins, of which at least MRP5 and MRP8 have been demonstrated to confer drug resistance to 5-FU [31,75]. This suggested role of MRP5 in mediating 5-FU chemoresistance was tested in pancreatic cancer cells by specific RNA interference silencing MRP5 expression. Indeed, such MRP5-silenced Capan-1 cells in which *MRP5* mRNA expression was downregulated to about 25% of that in control cells [26] showed an increased sensitivity to 5-FU as compared to parental or vector-transfected Capan-1 cells (Table 1) [26]. The contribution of MRP5 to 5-FU resistance was confirmed in a more recent study using Patu-02 pancreatic cancer cells where knock down of MRP5 also significantly increased cellular cytotoxicity of 5-FU [76].

### 2.4. Cellular Protein Pattern in 5-FU-Resistant Pancreatic Cancer Cells

**Figure 4.**
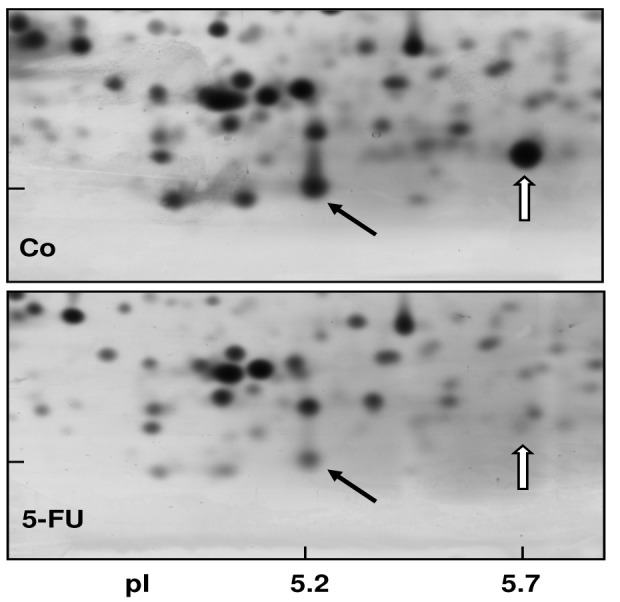
Loss of S100A4 protein in 5-FU resistant Capan-1 cells. Total cell lysates from parental Capan-1 cells (Co; *upper panel*) or 5-FU resistant Capan-1 cells (5-FU; *lower panel*) were subjected to 2D-DIGE analysis. *Black arrows:* S100A6, *white arrows:* S100A4 protein spot. Differentially expressed spots were matched and analyzed by a 2D-gel image analysis program and were identified by mass spectrometry.

**Figure 5.**
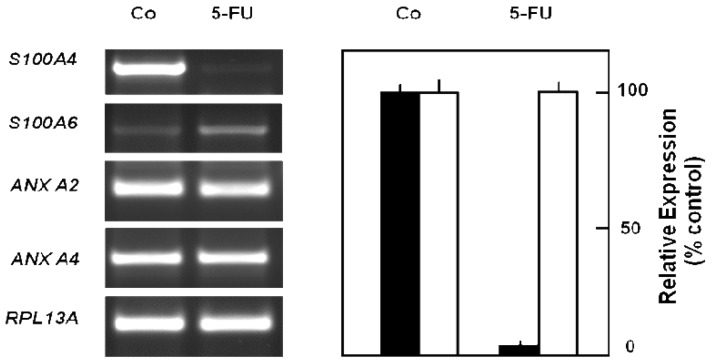
Loss of *S100A4* mRNA expression in 5-FU resistant Capan-1 cells. *Left panel:* Expression of *S100A4*, *S100A6*, *ANX A2*, *ANX A4* mRNAs were analyzed by RT-PCR (Image is a representative example of 2 biological, 2 technical replicates each). *Right panel: S100A4* (black columns) and *S100A6* mRNA expression (white columns) was quantified by QRT-PCR using *RPL13A* as housekeeping gene as in Figure 3; mean values from each condition with at least 2 biological and 2 technical replicates; bars indicate S.D. *Co:* Parental Capan-1 cells; *5-FU:* 5-FU resistant Capan-1 cells.

In order to obtain a direct fingerprint of cellular drug response, we analyzed the protein expression profiles of 5-FU resistant cells compared to parental pancreatic Capan-1 cancer cells by two-dimensional fluorescence difference gel electrophoresis (2D-DIGE). Among the approximately 1,200 separated proteins, the resistant cells showed altered expression of several proteins (cut-off: at least two-fold up-or down-regulated expression), for example, Annexin A2 and A4, and also the S100A6 protein spot which was diminished to about 30% of the amount in parental Capan-1 cells (Table 2). However, 5-FU resistant cells particularly showed massively reduced expression of a protein identified by ESI-MS/MS and confirmed by Western blot as the Ca-binding protein S100A4 (Figure 4). Interestingly, this S100A4 protein is discussed to have a role in cancer metastasis and chemoresistance [77-79]. Recently, S100A4 knockdown was reported to increase sensitivity of pancreatic cancer cells towards gemcitabine [80]. It remains to be clarified whether the apparently contradictory relation between S100A4 expression and chemoresistance is due to the different drugs used (5-FU, gemcitabine), different cell lines, or different experimental setups (acute toxicity *vs.* acquired resistance). Our novel finding of a loss of *S100A4* expression in 5-FU resistant cells was confirmed at the mRNA level (Figure 5). In contrast, the mRNA expression levels of *S100A6* and of two members of the annexin family (*ANX A2*, *ANX A4*) were demonstrated to be practically unaltered in 5-FU resistant Capan-1 cells (Figure 5). Further studies including RNA interference strategies are underway to clarify which direct or indirect role S100A4 may play in the 5-FU resistance phenotype of pancreatic cancer cells.

### 2.5. Transporter Expression and Resistance in Gemcitabine Treated Pancreatic Cancer Cells

**Figure 6.**
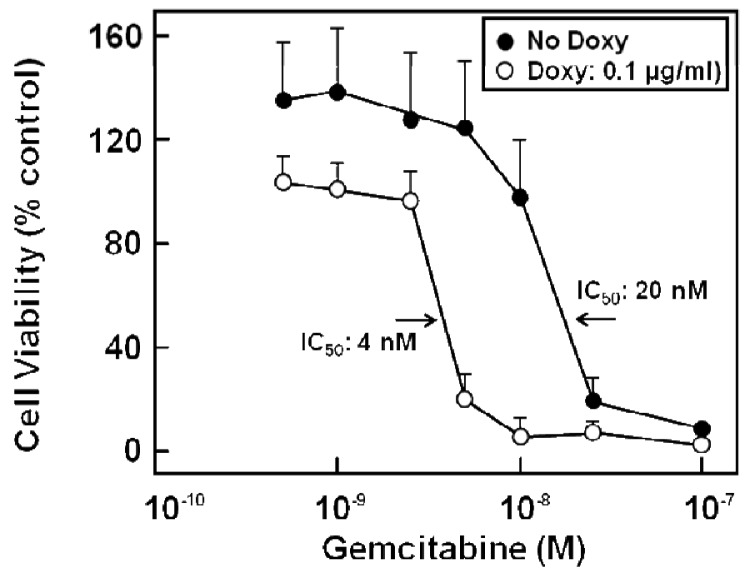
Gemcitabine cytotoxicity in parental and MRP5-silenced PANC-1 cells. PANC-1 cells containing doxycycline-inducible shRNA targeting *MRP5* mRNA (PANC-1/shMRP5 cells) were treated with doxycycline (100 ng/mL; *open columns*) or vehicle (*filled columns*) for 6 days and exposed to indicated gemcitabine concentrations for another 6 days before determination of cell viability. Mean values are from triplicate samples; bars indicate SD (adapted from [27]).

Various chemotherapy regimens using gemcitabine together with other drugs have been developed, some combining gemcitabine with 5-FU and resulting in improved benefit [81-83]. In addition, studies with human pancreatic cancer cells *in vitro* [39] or in a murine xenograft model [84] showed a better therapeutic effect when 5-FU was administered before gemcitabine. Furthermore, chemotherapeutic drug treatment of cells can alter the expression of nucleoside and ABC transporters [39,85], and drug export via MRP transporters contributes to cellular resistance against various chemotherapeutic compounds [23,25,26,28,30-32]. Indeed, gemcitabine alone or in combination with 5-FU treatment has been demonstrated to alter the expression of various MRPs, but also of uptake transporters of the CNT and ENT families [27]: While 5-FU or gemcitabine alone also elicited the enhanced expression of *MRP5* and *ENT1* mRNA, this upregulation was highest in all investigated pancreatic cancer cells after combined drug administration and was most prominent in Capan-1 and PANC-1 cells, with *MRP5* increasing 33-fold, and *ENT1* increasing 52-fold in Capan-1 cells [27]. Interestingly, the expression of the nucleoside transporter *CNT3* increased more than 100-fold in 5-FU/gemcitabine-treated Capan-1 cells [27]. In addition, MRP5 had been suggested to affect chemoresistance against gemcitabine [32,86], and this role of MRP5 in chemoresistance, which we earlier demonstrated for 5-FU [23], is evident also from results obtained with stably MRP5-silenced pancreatic cancer cells. In these experiments, *MRP5* mRNA expression was down to about 50% of controls as determined by QRT-PCR, and the cells possessed a markedly increased sensitivity to gemcitabine (Figure 6) [27].

## 3. Experimental Section

### 3.1. Cells and Drugs

Human pancreatic carcinoma cell lines (Capan-1, Capan-2, PANC-1, AsPC-1, BxPC-3, MiaPaCa-2, PaCa44) and immortalized pancreatic stellate cells (RLT-PSC) [67,87] were cultured at 37 °C, 5% CO_2_, and 95% humidity as reported [26]. Gemcitabine was obtained from Eli Lilly (Indianapolis, IN, U.S.), 5-FU from Teva (Kirchzarten, Germany), doxycycline from Sigma-Aldrich (St. Louis, MO, U.S.), and tetracycline-free fetal bovine serum from Clontech (Mountain View, CA, U.S.).

### 3.2. Generation of Resistant and Knockdown Cell Lines

Capan-1 cells with acquired resistance towards 5-FU (Capan-1/5-FU cells) were established as by adaptation of parental Capan-1 cells to increasing concentrations of 5-FU reaching 15.3 μM 5-FU (2 μg/mL) within 14 passages; these 5-FU resistant Capan-1 cells were subsequently maintained in medium containing 15.3 μM 5-FU. Silencing of endogenous MRP5 was achieved in two cell lines: in Capan-1 cells, RNA interference was performed using the BLOCK-iT™ Pol II miR RNAi expression vector kit (Invitrogen) with the pcDNA™6.2-GW/miR vector containing one of three miRNAs directed against different regions of the *MRP5* mRNA (base position 266–286, 330–350, or 433–453), which were stably transfected into Capan-1 cells using Fugene HD (Roche, Mannheim, Germany). For control, Capan-1 cells were transfected with pcDNA™6.2-GW/miR-neg vector (Invitrogen) expressing pre-miRNA, the mature miRNA of which does not target any known vertebrate gene. Stable clones were selected using blasticidin (5 μg/mL) and analyzed by RT-PCR, QRT-PCR, and agarose gel electrophoresis for their relative expression of *MRP5/RPL13A* mRNA as reported [26]. Targeted MRP5 knockdown in PANC-1 cells was achieved using the pSingle-tTS-shRNA vector (Clontech) containing one of three doxycycline-inducible short hairpin RNAs (shRNAs) targeting *MRP5* mRNA (oligos for bases 158–176 (target sense and antisense sequence in italics): 5′-TCGAGG-*CCGTGAAGATTCCAAGTTC*-TCAAGAGA-*GAACTTGGAATCTTCACGG*-CTTTTT TACGCGTA-3′ and 3′-CCGGCACTTCTAAGGTTCAAGAAGTTCTCTCTTGAACC-TTAGAAGTGCCGAAAAAATGCGCATTCGA-5′; for bases 368-386: 5′-TCGAGG-*AGCTCAGAATCC TGGATGA-*TTCAAGAGA-*TCATCCAGGATTCTGAGCT*-CTTTTTTACGCGT-A-3′ and 3′-CCTCGAGTCTTAGGACCT ACTAAGTTCTCTAGTAGGTCCTAAGACTC-GAGAAAAAATGCGCA TTCGA-5′; for bases 547–565: 5′-TCGAGG-*CTCTCAATGGAAGACGTGT*-TTCAAGAGA-*ACACGTCTTCCATTGA GAG*-CTTTTTTACGCGT-A-3′ and 3′-CCGAGAGTTACCTTCTGCACAAAGTTCTCTTGTGCAGAA GGTAACTCTCG-AAAAAATGCGCATTCGA-5′). Selected clones of these PANC- 1/shMRP5 cells were kept under geneticin (800 μg/mL) and were cultured in tetracycline-free medium. *MRP5* silencing was checked by QRT-PCR using PANC-1/shMRP5 clones treated without or with doxycycline for 3-6 days with replenishment of doxycycline every 48 h.

### 3.3. Drug Treatment of Cells

Cells were incubated with gemcitabine (20 μM) for 1 h, then medium was replaced with fresh medium containing no gemcitabine, and RNA was isolated 72 h later. In experiments comparing the effects of single or combined treatment of cells, the following schedules were applied: (a) 5-FU (30 μM, 24 h), then fresh drug-free medium; (b) gemcitabine (20 μM, 1 h), then fresh drug-free medium; (c) 5-FU (30 μM, 24 h), then gemcitabine (20 μM, 1 h), then fresh drug-free medium. RNA isolation was performed 4 days after 5-FU addition or 3 days after gemcitabine treatment.

### 3.4. Cytotoxicity Studies

Cytotoxicity of 5-FU was performed as reported [26] with at least triplicate biological and technical replicates for each condition. Cell viability was determined after 3 days of continued drug treatment using the CytoScan WST-1 cell cytotoxicity assay (G-Biosciences, St.Louis, MO, U.S.). For determination of gemcitabine cytotoxicity in stably MRP5-silenced PANC-1/shMRP5 cells, cells were treated for 6 days without or with doxycycline (100 ng/mL), trypsinized, seeded into 96-well plates (3,000 cells/well), and exposed to gemcitabine for another 6 days before WST-1 assay; during the whole experiment, doxycycline was replenished every 48 h.

### 3.5. RT-PCR and QRT-PCR Analyses

mRNAs were detected or quantified by RT-PCR or QRT-PCR, respectively, with these specific sense and anti-sense primers: *ENT1* (gene symbol: SLC29A1; accession: NM_004955), 5′ -AGTGGCTCGGAGCTATCAGA-3′ and 5′ GTGCTCGAA-GACCACAGTCA-3′ (588 bp fragment, bases 918-1505); *CNT3* (gene symbol: SLC28A3; accession: NM_022127), 5′ -ATGAATTCAGCCCTGTCCTG-3′ and 5′-AAACGTGATGGCAGT-TGATG-3′ (484 bp fragment, bases 1482-1965). *Annexin A2* (gene symbol: ANXA2; accession: NM_001002857), 5′ -AAAGTACGGCAAGTCCCTGT-3′ and 5′ -TTGGGGGTAATGCTAAC-GTC-3′ (223 bp fragment, bases 1063-1285); *Annexin A4* (gene symbol: ANXA4; accession: NM_001153), 5′-TGGAAAGTCTCTGTACTCGTTCAT-3′ and 5′-GCTTTGAAATGCA-AGTACAGCTT-3′ (294 bp fragment, bases 952-1245); *S100A4* (accession: NM_019554), 5′-CTTGCACACGCTGTTGCTAT-3′ and 5′-AACTTGCTCAGCATCAAGCA-3′ (467 bp fragment, bases 73-539); *S100A6* (accession: NM_014624), 5′-CGACCGCTATAAGGCCAGT-3′ and 5′- CCCACCACTGGATTTGACTC-3′ (437 bp fragment, bases 214-650); *OATP-A* (gene symbol: SLCO1A2; accession: NM_021094), 5′-CCCACATAGGATGTTGGTTATCC-3′ and 5′-ATGTATGTAATCCCACACCAAGG-3′ (495 bp fragment, bases 1163-1657); OATP-B (gene symbol: SLCO2B1; accession: NM_007256), 5′- CGACTCAACGTGCAGCCATC-3′ and 5′- CCGACACTAGCAATTGCTGCT-3′ (437 bp fragment, bases 1659-2095); OATP-C (gene symbol: *SLCO1B1;* accession: NM_006446), 5′ -TGCACTTGGAGGCACCTCAC-3′ and 5′-CTTCATCCATGACACTTCCATT-3′ (359 bp fragment, bases 1644-2002); OATP-D (gene symbol: SLCO3A1; accession: NM_013272), 5′-GTTGGGCTTCATCCCTCCAC-3′ and 5′-TTAGTCACTATAAAACGGACT-3′ (392 bp fragment, bases 1749-2140); OATP-E (gene symbol: SLCO4A1; accession: NM_016354), 5′-GAGACTGTAGCTGTATCCCTC-3′ and 5′-GCGGTGGTCAGACGCTGCT-3′ (531 bp fragment, bases 1646-2176); OATP-H (gene symbol: SLCO4C1; accession: NM_180991), 5′-CTCCTATAACTGTGTCTATCCT-3′ (395 bp fragment, bases 1787-2181); OATP-8 (gene symbol: SLCO1B3; accession: NM_019844), 5′ -TCATAAACTCTTTGTTCTCTGC-3′ and 5′-GTTGGCAGCAGCATTGTCTTG-3′ (482 bp fragment, bases 1625–2106). The mRNA expression analyses of *MRP* isoforms and of *RPL13A* were performed as reported [26].

### 3.6. DIGE and Mass Spectrometry Analyses

Parental Capan-1 and 5-FU resistant Capan-1 cells [26] were analyzed following lysis (9.5 M urea, 2% CHAPS, 0.8% Pharmalyte, 1% DTT and 5 mM Pefabloc SC plus) and centrifugation at 13000× *g* for 60 min at 15 °C. Protein labeling for DIGE was conducted as described in the Ettan DIGE user manual. 50 μg of each protein sample were labeled with 400 pmol of CyDye DIGE Fluor minimal dyes Cy2, Cy3, and Cy5. Internal standard for DIGE was generated by pooling parental and resistant Capan-1 cell samples. Individual parental and resistant Capan-1 cell samples were labeled with Cy3 or Cy5, while the internal standard was always labeled with Cy2. First dimensional electrophoresis was performed as reported [88] on linear immobilized pH gradient strips using 50 μg total protein/strip. The second dimension (SDS-PAGE) used 12.5% gels running for about 22 h in TGS electrode buffer at 10 °C and 85 V, and the protein spots were visualized on the Typhoon 9400 variable mode imager using optimal emission wavelength for each DIGE flours. DeCyder image analysis software was used to analyze the DIGE images as described in the Ettan DIGE user manual.

For mass spectrometric analysis, Coomassie stained gel pieces containing the proteins of interest were manually excised from a corresponding preparative gel and subjected to in-gel tryptic digestion [88]. Proteins were identified by nanoLC-ESI-MS/MS on an LCQ mass spectrometer as described [89].

## 4. Conclusions

In summary, since the overall benefit of radio- and chemotherapeutic treatment of pancreatic cancer is at present still regrettably low, the overview on the current experimental evidence of drug-induced alterations in relevant transporter expression and on the contribution of these specific cellular membrane factors towards chemoresistance stresses the importance of integrating the role of uptake and export transporters of the nucleoside and MRP families into strategies aimed at developing improved chemotherapies against pancreatic carcinoma. To this aim, experimental studies using *in vivo* models resembling human pancreatic cancer are required to proof a causal connection between expression and activity of transporters such as MRP5 and tumor resistance to 5-FU and gemcitabine. It will further be interesting to study in more detail the possible connection between drug transporters and cell adhesion-mediated drug resistance (CAM-DR) [5,8990] as well as cell signaling pathways such as the sonic hedgehog pathway, which has recently be demonstrated to contribute to gemcitabine resistance in mice *in vivo* [91]. Such *in vivo* studies will certainly also increase our understanding of the role of interacting mechanisms between pancreatic stellate cells and pancreatic cancer cells in chemoresistance (for review see [92]).

## Figures and Tables

**Table 1.** Modulation of 5-FU cytotoxicity by MRP5 silencing. Capan-1 cells stably transfected with control vector (*Controls*) or with vector containing pre-miRNA specifically silencing MRP5 (*MRP5-silenced cells*) were subjected to 5-FU at indicated concentrations for 72 h before cytotoxicity determination. Mean values are from triplicate technical samples [26].

**5-FU (μg/ml)**	**Cytotoxicity (%) ± S.D.**	

	**Controls**	**MRP5-silenced Cells**
0	0	0
0.06	0.6 ± 0.7	43.8 ± 0.9
0.13	3.5 ± 6.3	44.9 ± 0.7
0.25	11.2 ± 3.6	43.2 ± 0.7
0.50	21.3 ± 3.2	48.6 ± 2.0

**Table 2.** Proteins with increased (up) or decreased (down) expression in 5-FU resistant Capan-1 cells as compared to parental Capan-1 cells.

**Protein Name**	**Acc. No.**	**Expression**	**Peptides Matched**	**Sequence Coverage**	**MW (th./exp.)**	**pi (th./exp.)**
Cathepsin D (heavy chain)	4503143	only in 5-FU resistant cells	5	12%	26.7/30.8	5.5/5.2
Glyoxalase I	15030212	2-fold up	6	22%	21.0/24.7	5.1/5.1
Isocitrate dehydrogenase 3 /alpha enolase	48256839	2-fold up	6	17%	40.0/37.6	6.4/6.0
Tropomyosin 3	24119203	2-fold up	7	23%	29.2/31.1	4.7/4.7
Peroxiredoxin 3	32483377	2-fold up	6 a	44%	26.1/26.7	7.0/6.2
26S Protease reg. subunit 6A	20532406	2-fold down	4	12%	49.4/47.2	5.1/5.2
Annexin A2 (fragment)	16306978	3-fold down	13	41%	38.8/28.9	7.6/5.6
Cofilin 1	5031635	down (only in parental cells)	7	48%	18.7/14.6	8.2/5.7
Dynactin 2	5453629	3-fold down	5	19%	44.9/50.2	5.1/5.2
Gelsolin-like capping protein	63252913	3-fold down	4	21%	38.8/39.4	5.8/6.0
Keratin 19 (4 spots)	24234699	3 fold down	23	53%	44.1/40.0	4.9/4.9–5.1
Lectin, galactose-bindg., soluble	4504981	3 fold down	4	33%	15.0/15.0	5.3/5.1
S100A4 (Calvasculin)	4506765	down (only in parental cells)	2 b	19.8%	11.7/12.7	5.8/5.7
S100 A6 (Calcyclin)	30582769	3-fold down	2 b	16%	10.2/11.9	5.3/5.2
S100A11 (Calgizzarin)	5032057	3-fold down	4	33%	11.8/13.4	6.6/6.1
Stratifin; 14-3-3 sigma	5454052	2-fold down	7 b	26%	27.9/29.2	4.7/4.6
Triosephosphate isomerase 1	4507645	2-fold down	6	29%	26.9/28.3	6.5/6.3
UMP-CMP kinase	12644008	2-fold down	1 a		22.2/24.2	5.4/5.7

MW: molecular weight (kDa); th.: theoretical; exp.: experimental; pI: isoelectric point.

a confirmed by Post-Source Decay (PSD) MALDI-MS;

b identified by ESI-MS/MS.
